# Synergistic Flow Field and Ion–Dipole Interactions Enable γ-β Phase Transformation in Poly(vinylidene fluoride)

**DOI:** 10.3390/polym18151901

**Published:** 2026-08-03

**Authors:** Qian Wang, Hong-Biao Yin, Hua-Jian Li, Xiang Bai, Fei Wang, Jianguo Liang, Guo-Zhen Ma, Jia-Yi Ren, Zhanchun Chen

**Affiliations:** 1 College of Mechanical Engineering, Taiyuan University of Technology, Taiyuan 030024, China; 2 Shanxi Energy Internet Research Institute, Taiyuan 030024, China

**Keywords:** PVDF, flow field, solid-phase extrusion, ion–dipole interactions, polar phases

## Abstract

Poly(vinylidene fluoride) (PVDF) exhibits excellent piezoelectric properties governed by the content and orientation of its polar phases. Herein, a synergistic regulation strategy integrating a flow field induced by a designed solid-phase extrusion die and ion–dipole interactions introduced by CTAB is established to achieve highly oriented β phase in PVDF. The incorporation of CTAB promotes the formation of the γ phase before extrusion, providing a structurally favorable precursor for the subsequent flow-induced γ-β phase transformation. During solid-phase extrusion, the converging flow field drives extensive molecular chain alignment, promoting the γ-β phase transformation and substantially enhancing both β phase content and orientation. The synergistic effects of CTAB-induced ion–dipole interactions and the converging flow field further regulate the melting behavior and crystal perfection of PVDF, driving the transformation of the lamellar structure into highly oriented lamellar bundles along the extrusion direction. Among all compositions studied, the blends containing 5 wt% CTAB exhibit the optimal polar phase content and orientation characteristics, along with the highest dielectric constant. This work elucidates the synergistic regulation of PVDF hierarchical structures by ion–dipole interactions and flow fields, offering an effective strategy for fabricating high-performance piezoelectric PVDF materials.

## 1. Introduction

Poly(vinylidene fluoride) (PVDF) is a semi-crystalline fluoropolymer renowned for its outstanding piezoelectric, ferroelectric, and pyroelectric properties [1,2,3]. The piezoelectric response of PVDF originates from the directional alignment of C-F dipoles within its polar phases. Owing to its flexibility, chemical inertness, and ease of processing, PVDF has demonstrated application prospects in flexible sensors, energy harvesters, self-powered devices, and biomedical monitoring [4,5,6,7].

The crystalline polymorphism of PVDF strongly influences its functional characteristics. Currently, PVDF exhibits five crystalline polymorphs, of which the α, β, and γ phases are the most common. The nonpolar α phase adopts a TGTG’ conformation and is the thermodynamically stable product of melt crystallization. The polar β phase adopts an all-trans TTTT conformation and delivers the highest piezoelectric performance because its all-trans chain conformation aligns the C-F bond dipoles in the same direction, producing the maximum net dipole moment of approximately 7 × 10^−30^ C·m [8,9,10]. The polar γ phase adopts a T_3_GT_3_G’ conformation. Its piezoelectric activity is lower because periodic gauche defects reduce dipole alignment along the polymer chain. Nevertheless, the γ phase possesses approximately 40% higher breakdown strength (≈500 kV/mm) and a higher Curie temperature than the β phase, rendering it uniquely suitable for high-temperature piezoelectric applications [11,12,13,14]. PVDF exhibits several phase transformation pathways, including α-β phase transformation through mechanical stretching, α-γ phase transformation through high-temperature annealing or additive-induced crystallization, and γ-β phase transformation through mechanical deformation or high-electric-field poling. These transformations have been widely studied to elucidate their mechanisms and guide phase regulation [15,16,17,18].

Among the various strategies for inducing polar phases in PVDF, exploiting ion–dipole interactions between ionic additives and PVDF chain segments offers distinct practical advantages, owing to its simplicity and the elimination of complex post-treatments [19,20]. As a cationic quaternary ammonium surfactant, CTAB forms strong ion–dipole interactions between its quaternary ammonium headgroups and the -CF_2_ groups of PVDF. These interactions promote the conformational transition toward the all-trans state and facilitate the nucleation of polar phases [21,22]. Recent studies on ionic liquid-assisted phase transformations in PVDF have corroborated the critical role of ion–dipole interactions in inducing polar phases. Imidazolium-based ionic liquids promote the α-γ phase transformation through ion–dipole interactions with the -CF_2_ dipoles [23]. For the PVDF/CTAB blend system, previous studies have demonstrated that the addition of only 1 wt% CTAB can increase the polar phase content to 97.6% [24]. Further studies revealed that a γ phase is preferentially formed during melt blending, whereas direct crystallization into 95.3% β phase is achieved under coating conditions, suggesting that the interfacial environment of the ion–dipole interaction determines the specific type of polar phase selected [25]. Nevertheless, these studies were conducted under quiescent conditions, which typically yield randomly oriented lamellar structures, leaving the potential of combining ion–dipole interactions with flow-induced orientation largely unexplored.

The application of a strong flow field, particularly during solid-phase processing, is a key route for manipulating hierarchical polymer structures. The flow field drives molecular chain extension and orientation along the processing direction through significant plastic deformation of the polymer matrix, generating characteristic crystal polymorphs and highly oriented morphologies distinct from those produced under quiescent conditions [26,27]. In PVDF systems, the type and concentration of extrinsic ions can significantly alter the polymorph selection and nucleation rates under flow fields [28]. Moreover, strain rate and temperature profoundly influence the flow-induced phase selection of PVDF, where a pure β phase can be directly generated at a strain rate of 10 s^−1^ within a temperature window of 155–170 °C [29]. Crucially, the degree of segmental orientation dictates the formation of polar phases, with the β phase formation requiring a high degree of chain extension, whereas γ phase formation can be triggered at relatively low chain orientation levels. The presence of pre-existing γ phase promotes the γ-β phase transformation during deformation, providing a pathway for tailoring the polymorphic evolution and crystalline structure of PVDF [30,31].

Solid-phase extrusion (SPE), as a semi-solid processing of polymers, provides such a route by imposing pressure-driven plastic deformation through a converging die, enabling the formation of highly oriented structures during solid-phase processing. This process exerts a more rigorous fragmentation and rearrangement effect on the original crystalline structures, which is uniquely significant for fabricating highly oriented piezoelectric films [32,33,34]. Additionally, in multicomponent PVDF blends, introducing a secondary component concurrently influences the crystallization behavior and crystalline polymorphism of PVDF [35,36]. However, the synergistic effect of ionic additives, particularly CTAB, and the converging flow field under SPE conditions remains systematically unexplored. The mechanism linking molecular conformations to mesoscopic morphologies also remains unclear.

Against this background, this study investigates PVDF/CTAB blends using a custom-designed SPE apparatus (Figure S1). First, a specific thermal history under pressure was applied to regulate the initial crystallization tendency and preferentially induce the γ phase. Subsequently, SPE was performed through the converging die at 130 °C to exploit the strong flow field for enhancing the β phase formation tendency and segmental orientation, thereby achieving the γ-β phase transformation in a single step. A comparative experimental design was employed, comprising the die inlet samples (without die extrusion) and the extruded samples. The structural evolution of PVDF/CTAB blends was systematically elucidated across multiple structural levels, from molecular conformations and crystalline phases to lamellar organization and microscopic morphology. This work elucidates the synergistic effects of CTAB-induced ion–dipole interactions and converging flow during SPE on the polymorphic evolution of PVDF.

## 2. Materials and Methods

### 2.1. Materials

Poly(vinylidene fluoride) (PVDF, Kynar^®^ 720, Arkema Inc., King of Prussia, PA, USA)., with a melt flow index (MFI) of 24 g/10 min measured at 230 °C under a 5 kg load. Cetyltrimethylammonium bromide (CTAB, Shanghai Macklin Biochemical Co., Ltd., Shanghai, China) was purchased and used as received.

### 2.2. Sample Preparation

First, the raw PVDF was dried at 80 °C for 48 h to remove residual moisture. Subsequently, PVDF was melt-blended with various weight fractions of CTAB (1, 3, 5, and 10 wt%) using a co-rotating twin-screw extruder at a die temperature of 200 °C and a screw speed of 150 rpm. The extruded strands were pelletized and then dried in a vacuum oven at 80 °C for 36 h before further processing. Neat PVDF was also processed via twin-screw extrusion under identical conditions, serving as the control. Additionally, sample consolidation and SPE were conducted using a custom-designed SPE apparatus developed by our research group.

**Figure 1:**
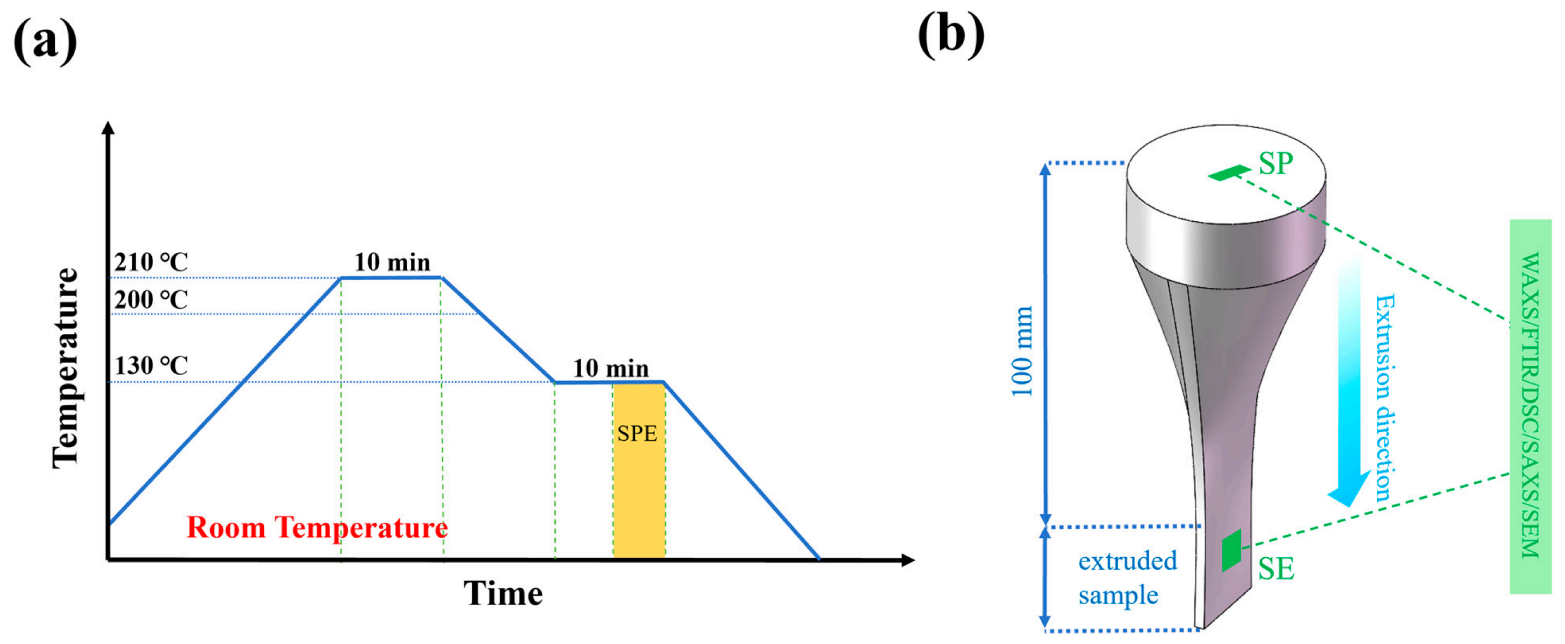
Schematic of the solid-phase extrusion process and apparatus. (**a**) Temperature–time profile of the experimental process. (**b**) Schematic illustration of the converging die used in solid-phase extrusion, showing the inlet (SP) and extruded (SE) sample locations, together with the corresponding characterization methods.

The experimental procedure is schematically illustrated in Figure 1a. The granulated PVDF/CTAB blends were loaded into the extrusion chamber, heated to 210 °C, and held isothermally for 10 min to erase thermal history. Subsequently, a pressure of 10 MPa was applied for consolidation. The system was then cooled to the extrusion temperature of 130 °C and maintained for 5 min to ensure thermal equilibration before SPE, yielding the final samples. The sample nomenclature is defined as follows: The extruded PVDF films containing 1, 3, 5, and 10 wt% CTAB are designated as SE-C1, SE-C3, SE-C5, and SE-C10, respectively, while the neat extruded PVDF film is labeled as SE-N. Their corresponding die inlet counterparts (which underwent the identical thermal and consolidation history but were not subjected to die extrusion) are denoted as SP-C1, SP-C3, SP-C5, SP-C10, and SP-N, and serve as references for comparative analysis in the Results and Discussion section.

### 2.3. Characterization Methods

Two-dimensional wide-angle X-ray diffraction (2D-WAXD) and two-dimensional small-angle X-ray scattering (2D-SAXS) measurements were performed at beamline BL16B of the Shanghai Synchrotron Radiation Facility (SSRF, Shanghai, China) using an X-ray wavelength of 0.124 nm. Data were collected in transmission geometry using a Pilatus M detector (1475 × 1679 pixels; pixel size: 172 × 172 μm^2^, DECTRIS AG, Baden-Daettwil, Switzerland). For 2D-WAXD, the sample-to-detector distance was 231.3 mm, and the X-ray beam penetrated the entire film thickness, enabling characterization of the bulk crystalline structure. The 2D-WAXD patterns were integrated over the full range (0–360°) to obtain the corresponding 1D-WAXD profiles. For 2D-SAXS, the sample-to-detector distance was 2344.2 mm, and one-dimensional SAXS profiles of scattering intensity (*I*) as a function of scattering vector (*q*) were obtained by sector integration of the 2D-SAXS patterns over an azimuthal angle range of 255–285°. The crystalline (*L*_c_) and amorphous (*L*_a_) layer thicknesses were obtained from the one-dimensional electron density correlation function *K*(z) [37], calculated by Fourier transform of *I*(*q*) (Equation (1)):

(1) $$K(z)=\frac{\int_{0}^{\infty }I(q)\cos(qz)dq}{\int_{0}^{\infty }I(q)dq}$$

where *I*(*q*) is the scattering intensity and *z* represents the real-space distance.

The surface crystalline structure of the films was characterized using a Fourier-transform infrared (FTIR) spectrometer (Nicolet 6700, Thermo Fisher Scientific, Waltham, MA, USA) in the attenuated total reflection (ATR) mode. The scanning range was 4000–600 cm^−1^ with a resolution of 4 cm^−1^. Based on the FTIR spectra, the relative fractions of the different crystalline phases were quantified using the following method.

First, the fraction of the nonpolar α phase (*F*_α_) and the combined fraction of the polar β and γ phases (*F*_β,γ_) were determined according to the Beer–Lambert law [8]:

(2) $$F_{\alpha }=\frac{A_{763}}{A_{763}+\left(\frac{K_{763}}{K_{840}}\right)A_{840}}\times 100\%$$

(3) $$F_{\beta ,\gamma }=100\%-F_{\alpha }$$

where *A*_763_ and *A*_840_ denote the absorbance intensities of the α phase at 763 cm^−1^ and the combined β/γ phases at 840 cm^−1^, respectively. The term *K*_763_/*K*_840_ represents the ratio of the absorption coefficients at the corresponding wavenumbers.

Subsequently, the individual fractions of the β phase (*F*_β_) and γ phase (*F*_γ_) were resolved using their unique characteristic absorption bands at 1276 and 1234 cm^−1^, respectively:

(4) $$F_{\beta }=F_{\beta ,\gamma }\times \frac{A_{1276}}{A_{1276}+A_{1234}}$$

(5) $$F_{\gamma }=F_{\beta ,\gamma }\times \frac{A_{1234}}{A_{1276}+A_{1234}}$$

where *A*_1276_ and *A*_1234_ are the absorbance intensities at 1276 cm^−1^ and 1234 cm^−1^, respectively.

The melting behavior was investigated by differential scanning calorimetry (DSC; TA Q2000, TA Instruments, New Castle, DE, USA). Approximately 5 mg of each sample was heated from 40 °C to 210 °C at a heating rate of 10 °C/min under a nitrogen atmosphere.

The crystalline morphology was examined using field-emission scanning electron microscopy (FE-SEM; Inspect F, FEI, Eindhoven, Netherlands) at an accelerating voltage of 3 kV in secondary-electron imaging mode. For sample preparation, the specimens were carefully sectioned with a razor blade to expose a fresh surface and subsequently etched at 90 °C for 72 h using a chemical reagent prepared according to a previously reported method [38]. After thorough washing and drying, the fractured cross sections were sputter-coated with a thin gold layer to ensure electrical conductivity before imaging.

The dielectric constant and dielectric loss of the extruded samples were measured as a function of frequency (10^2^ to 10^6^ Hz) using a Tonghui TH2829C LCR meter equipped with a TH2829C-EPC3008 dielectric material test fixture (Changzhou Tonghui Electronic Co., Ltd., Changzhou, China), with the films placed between two circular electrodes for the dielectric measurements.

## 3. Results and Discussion

### 3.1. Crystalline Structure and Molecular Conformational Evolution of PVDF/CTAB Blends

The crystalline structure and molecular conformation of PVDF were characterized using a combination of WAXD and FTIR techniques. Specifically, WAXD provides statistical and macroscopic information on the bulk crystalline phase composition, while FTIR reveals the localized structural details of chain segment conformations within the surface layer.

**Figure 2:**
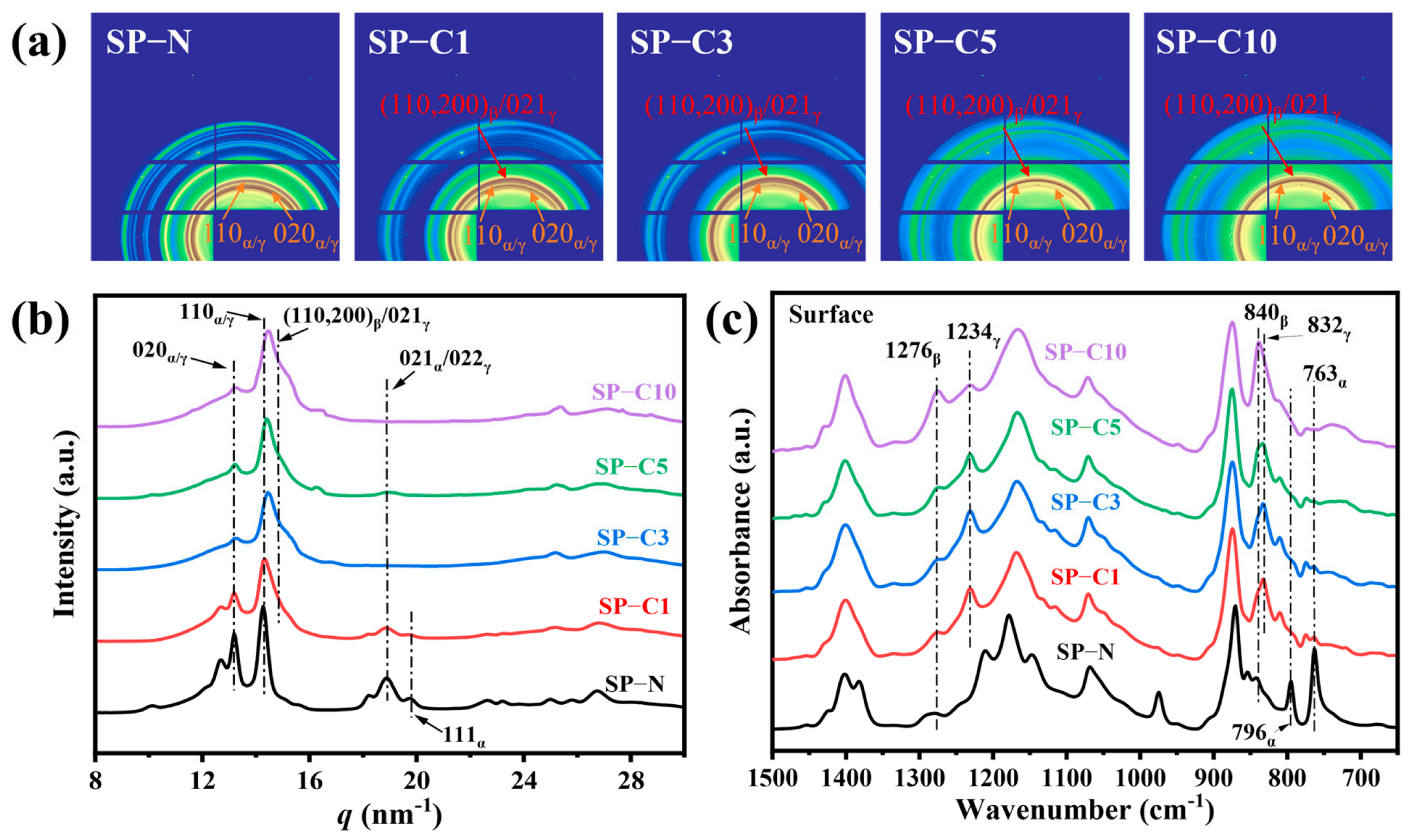
(**a**) 2D-WAXD patterns, (**b**) 1D-WAXD profiles, and (**c**) FTIR spectra of the die inlet PVDF/CTAB blends. The arrows indicate the characteristic diffraction peaks or absorption bands of different crystalline phases. Different colors represent different samples (SP-N, SP-C1, SP-C3, SP-C5, and SP-C10).

Figure 2a displays the 2D-WAXD patterns of the die inlet samples. For the neat sample (SP-N), only the characteristic diffraction rings of the α phase are observed, with no discernible polar phase reflections. Upon CTAB incorporation, a new diffraction ring corresponding to the overlapping β_(110,200)_/γ_(021)_ reflection becomes visible, and its progressive intensity strengthens with increasing CTAB content. Meanwhile, the α/γ_(110)_ and α/γ_(020)_ diffraction rings persist across all compositions, though their relative intensities evolve with CTAB content. Throughout, all diffraction rings remain continuous and isotropic.

Figure 2b shows the corresponding 1D-WAXD profiles, which further reveal the evolution of crystalline phases. For the neat sample, diffraction peaks are observed at *q* ≈ 13.04, 14.25, 18.83, and 19.69 nm^−1^, corresponding to the α_(020)_, α_(110)_, α_(021)_/γ_(022)_, and α_(111)_ planes, respectively (Table S1), confirming that neat PVDF remains dominated by the thermodynamically stable nonpolar α phase at the die inlet. With increasing CTAB content, the overlapping β_(110,200)_/γ_(021)_ reflections near *q* ≈ 14.74 nm^−1^ progressively strengthen and gradually become the dominant reflection, while the intensities of the overlapping α/γ_(020)_ and α_(021)_/γ_(022)_ reflections show an overall decrease. These results demonstrate that CTAB promotes the formation of polar phases in PVDF. The sharp α phase contribution within the overlapping α/γ reflections is progressively replaced by the broader γ phase contribution, resulting in a pronounced evolution of the overlapping reflections. Furthermore, the progressive shift of the α/γ_(110)_ reflection toward higher *q* values, consistent with the different lattice spacings of the α and γ phases, together with the disappearance of the α_(111)_ reflection, provides evidence for preferential stabilization of the γ phase and suppression of the α phase upon CTAB incorporation.

FTIR analysis further confirms the presence of the polar phases in the die inlet samples. It should be noted that the ATR-FTIR results mainly represent the molecular structure of the near-surface region of the die inlet samples. Neat PVDF exhibits prominent α phase absorption peaks at 763 and 796 cm^−1^, indicating that its chain segments predominantly adopt the TGTG’ conformation. Compared with neat PVDF, the incorporation of CTAB effectively promotes the formation of polar phases. At a low CTAB content of 1 wt%, polar phases are readily induced, with a dominant contribution from the γ phase. With increasing CTAB content to 3 wt%, the γ phase fraction further increases. Upon further addition of CTAB (≥5 wt%), the β phase content gradually increases, accompanied by a reduction in the γ phase. At a high CTAB loading of 10 wt%, the β phase becomes the dominant polar phase, while a considerable fraction of γ phase remains, suggesting the coexistence of β and γ phases [39]. Quantitative phase fraction analysis based on Equations (2)–(5) (Figure S2) corroborates these qualitative trends, showing that in the die inlet samples, the γ phase fraction reaches a maximum at 5 wt% CTAB before declining at 10 wt%. Moreover, the high compressive stress continuously applied by the plunger during the extrusion process (up to 300 MPa) may induce localized molecular chain rearrangement, contributing to the slight increase in β phase content detected on the surface of the die inlet samples by ATR-FTIR analysis.

**Figure 3:**
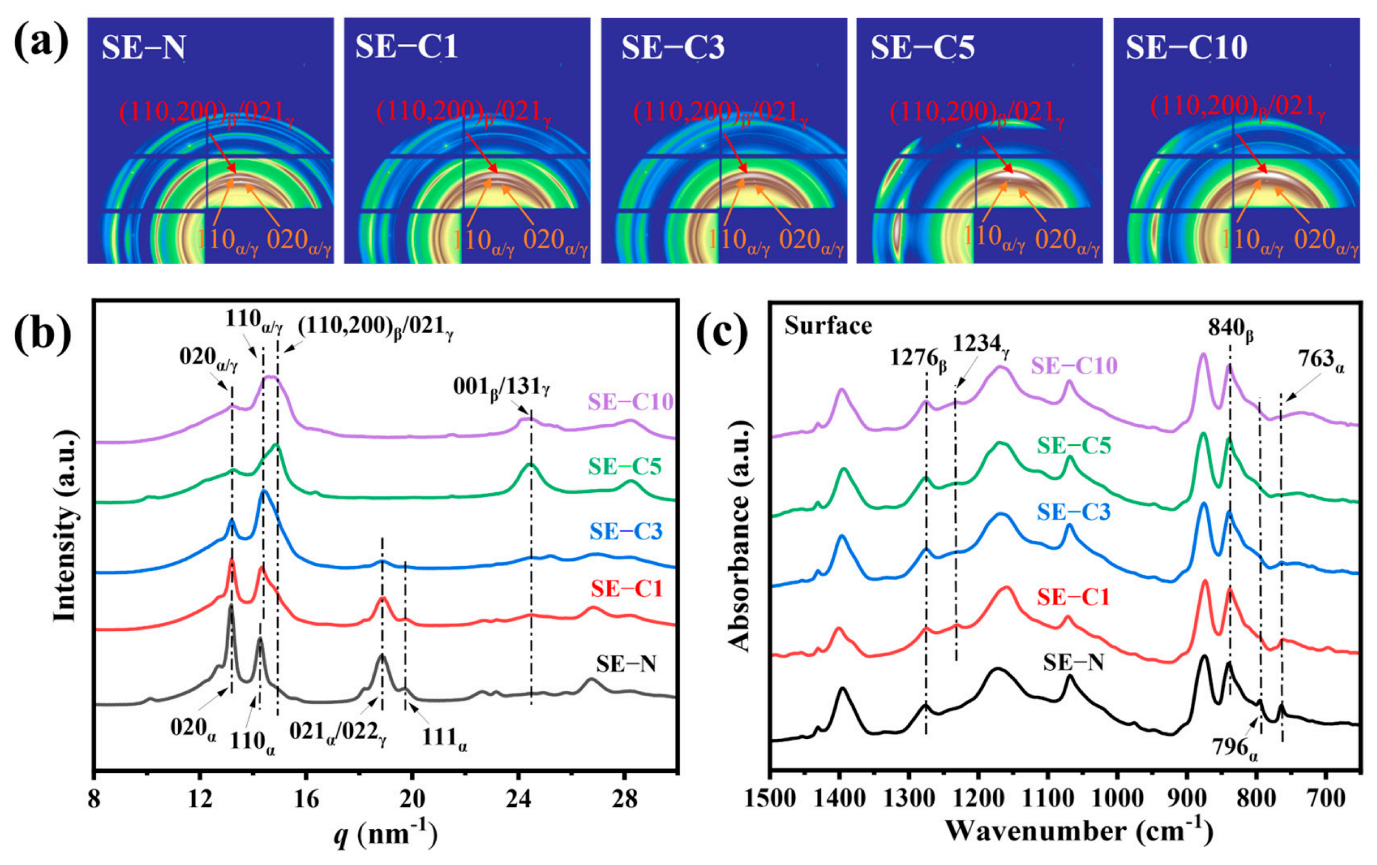
(**a**) 2D-WAXD patterns, (**b**) 1D-WAXD profiles, and (**c**) ATR-FTIR spectra of the solid-phase extruded PVDF/CTAB blends. The arrows indicate the characteristic diffraction peaks or absorption bands of different crystalline phases. Different colors represent different samples (SP-N, SP-C1, SP-C3, SP-C5, and SP-C10).

As shown in Figure 3a, comparison between the die inlet sample and the extruded neat PVDF sample (SE-N) highlights the effect of the converging flow field during SPE. After extrusion, the original continuous diffraction rings transform into partially developed arcs concentrated at specific azimuthal angles, indicating the formation of a pronounced crystalline orientation induced by flow deformation.

Figure 3b shows the 1D-WAXD profiles of the extruded samples. For the neat extruded sample (SE-N), compared with its die inlet counterpart, the overlapping β_(110,200)_/γ_(021)_ reflection shows a slight but discernible increase, indicating that the converging flow field alone can drive a limited degree of α-β phase transformation even in the absence of CTAB [40]. Meanwhile, the α phase reflections remain prominent, suggesting that a considerable fraction of α phase is preserved after SPE. The enhanced intensity of the α_(020)_ reflection is mainly associated with flow-induced preferred orientation of α phase rather than an increase in content of α phase. Upon CTAB incorporation, the crystalline evolution under identical extrusion conditions follows a distinct pathway. Compared with the extruded neat PVDF sample, all CTAB-containing extruded samples exhibit significantly enhanced β_(110,200)_/γ_(021)_ and β_(001)_/γ_(131)_ reflections, accompanied by a progressive suppression of α phase-related peaks. With increasing CTAB content, the intensity of polar phase reflections increases and reaches a maximum at 5 wt%, while a slight decrease is observed at 10 wt%, indicating a composition-dependent balance between ion–dipole interactions and flow-induced crystallization during extrusion.

ATR-FTIR spectra (Figure 3c) provide complementary information on the near-surface crystalline conformation. Compared with the die inlet samples, all extruded samples show weakened α phase absorption bands at 763 and 796 cm^−1^ and enhanced β phase bands at 840 and 1276 cm^−1^, suggesting the formation of a β-enriched near-surface region after extrusion. Notably, since ATR-FTIR is sensitive to the near-surface region, whereas WAXD reflects bulk-averaged information, the combined results suggest a possible depth-dependent distribution of crystalline phases.

### 3.2. Thermal Behavior and Polymorphic Melting Analysis of PVDF/CTAB Blends

**Figure 4:**
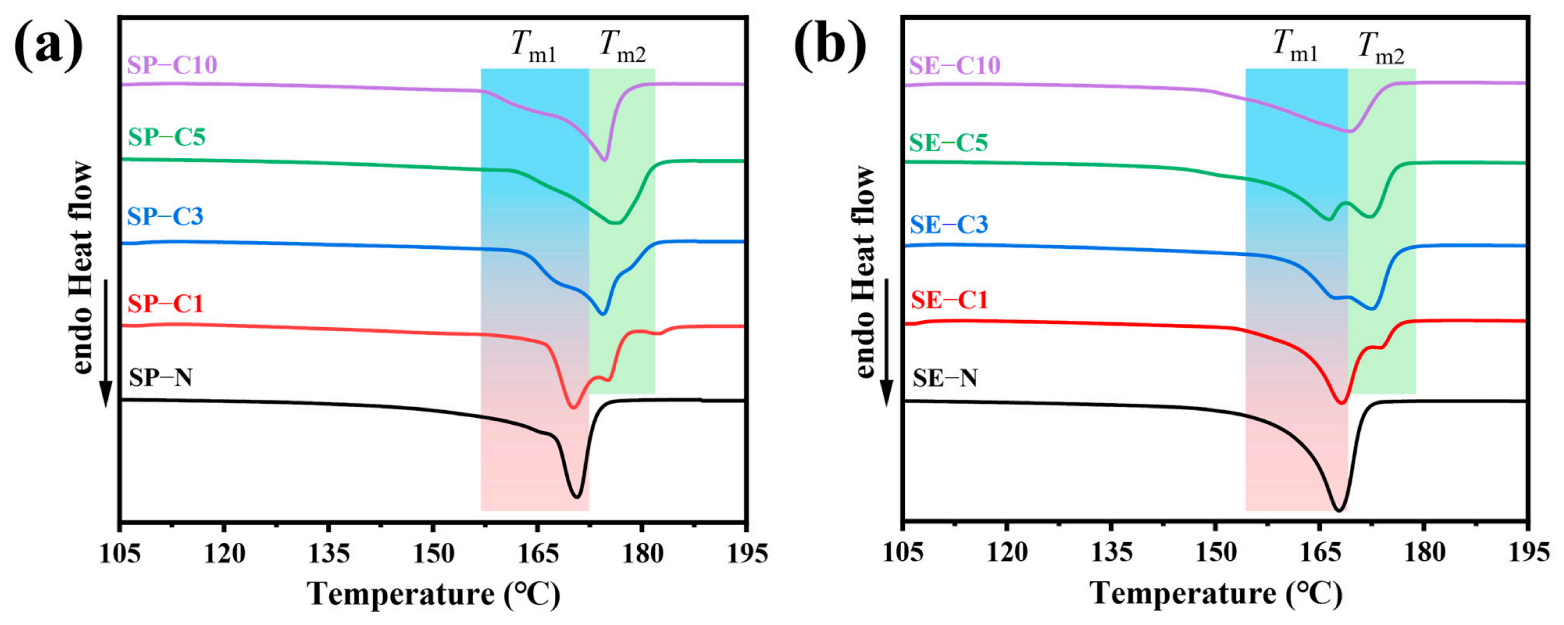
DSC first-heating thermograms of PVDF/CTAB blends with different CTAB contents. (**a**) Die inlet samples and (**b**) solid-phase extruded samples. The shaded regions indicate the temperature ranges corresponding to the two melting peaks(*T*_m1_ and *T*_m2_).

To further evaluate the influence of polymorphic evolution on the thermal behavior of PVDF, the melting behavior of the samples was characterized by DSC, and the corresponding first heating DSC curves and melting temperatures are shown in Figure 4 and Table 1. The melting endotherms can be broadly separated into two partially overlapping regions, including a lower-temperature melting peak (*T*_m1_, 163.2–170.7 °C) and a higher-temperature melting peak (*T*_m2_, 172.2–176.2 °C). Since the melting behavior of PVDF is related to its crystalline structure and degree of crystal perfection, the variation in melting peaks should be interpreted together with the structural evolution of the samples.

As shown in Figure 4a, the neat die inlet PVDF exhibits a dominant melting peak centered at approximately 170.7 °C, which is mainly attributed to the melting of α phase. Upon CTAB incorporation, the double-melting behavior becomes increasingly pronounced, accompanied by an enhanced high-temperature melting peak associated with the increased fraction of polar γ/β phases. This result agrees well with the WAXD and FTIR analyses. Considering the higher melting temperature of γ phase compared with α phase, the high-temperature melting peak is mainly attributed to γ phase, with a possible contribution from β phase [11,41,42,43,44,45]. With increasing CTAB content, the enhanced high-temperature melting contribution indicates the gradual increase of thermally stable polar phases.

The first heating DSC curves of the extruded samples are shown in Figure 4b, and the corresponding melting temperatures are summarized in Table 1. After SPE, the melting temperatures of the neat sample (*T*_m1_) and the CTAB-containing samples (*T*_m1_ and *T*_m2_) decrease by approximately 2–4 °C relative to their die inlet counterparts. This reduction is attributed to decreased crystalline perfection resulting from flow field-induced spherulitic fragmentation and lamellar rearrangement. Upon CTAB incorporation, the extruded samples retain clear double-melting characteristics. Notably, the low-temperature melting peak becomes more pronounced after extrusion. Given the marked increase in β phase fraction confirmed by WAXD and FTIR, the intensified low-temperature melting peak is therefore attributed to the newly formed β phase produced by the flow-induced γ-β phase transformation. The lower melting temperature indicates reduced crystalline perfection resulting from incomplete molecular chain rearrangement during SPE. Meanwhile, the high-temperature peak (*T*_m2_) retains appreciable intensity, suggesting that a portion of the pre-existing polar phases with higher crystalline perfection is preserved during SPE and contributes to the high-temperature melting peak.

Overall, the DSC results demonstrate that CTAB incorporation promotes polar phase formation, while converging flow-induced polymorphic transformation, together governing the melting behavior of PVDF. The double-melting characteristics indicate the coexistence of crystalline domains with distinct polymorphs and structural perfection.

### 3.3. Lamellar Structure of PVDF/CTAB Blends

**Figure 5:**
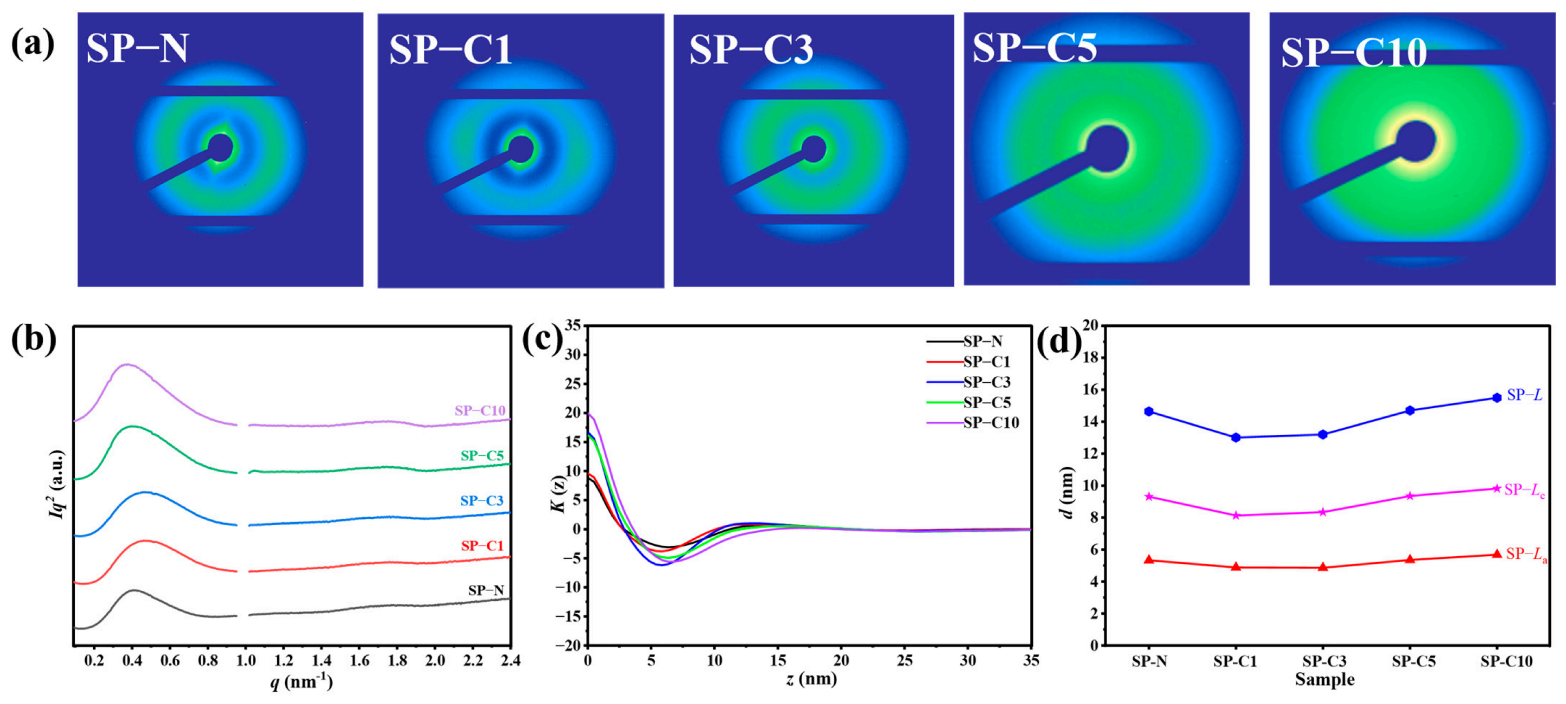
SAXS analysis of PVDF/CTAB samples at the die inlet. (**a**) 2D-SAXS patterns of the die inlet samples with different CTAB contents. (**b**) Corresponding 1D-SAXS profiles. (**c**) One-dimensional electron density correlation functions. (**d**) Comparison of the calculated lamellar structural parameters, including crystalline lamellar thickness (*L*_c_), amorphous layer thickness (*L*_a_), and long period (*L*) for the die inlet samples. Different colors represent different samples (SP-N, SP-C1, SP-C3, SP-C5, and SP-C10).

To further understand how CTAB and SPE synergistically induce the hierarchical structural evolution of PVDF, the lamellar structures of the die inlet and extruded samples were investigated by SAXS technology and one-dimensional electron density correlation analysis. The SAXS results provide information regarding the structural evolution of crystalline and amorphous regions at the mesoscale, complementing the phase evolution revealed by WAXD and FTIR. Figure 5a displays the 2D-SAXS patterns of the die inlet samples (without die extrusion). All die inlet samples exhibit nearly isotropic, circular scattering rings. This indicates that the lamellar structure exhibits a randomly oriented distribution in the absence of the forced converging flow field. As the CTAB content increases, the scattering intensity gradually increases, suggesting enhanced electron density contrast between crystalline and amorphous regions and improved local lamellar ordering.

The corresponding 1D-SAXS curves are displayed in Figure 5b. The die inlet samples all exhibit a broad scattering peak near *q* ≈ 0.45 nm^−1^ (*L* ≈ 14 nm). At low CTAB loading, the maximum scattering shifts slightly toward higher *q* values, corresponding to a reduced long period. With further increasing CTAB content, the peak gradually shifts back toward lower *q* values, indicating a subsequent increase in lamellar periodicity.

To determine whether this variation in long period originates mainly from the crystalline lamellae or the amorphous layer, one-dimensional electron density correlation functions are calculated from the Lorentz-corrected SAXS profiles according to the method described in Supplementary Materials Section S1.4. The quantitative parameters derived from these correlation functions are shown in Figure 5c and summarized in Figure 5d. Since *L*_c_ varies consistently with *L* while *L*_a_ remains nearly unchanged, the variation in long period primarily originates from changes in crystalline lamellar thickness. At low CTAB content, *L* decreases from 14.64 nm (SP-N) to 13.01 nm (SP-C1), accompanied by a comparable decrease in *L*_c_ from 9.31 nm to 8.13 nm. With further increasing CTAB content, both *L* and *L*_c_ gradually increase, reaching 15.5 nm and 9.82 nm for SP-C10, respectively. At low CTAB loading, enhanced heterogeneous nucleation increases the nucleation density, producing thinner and more refined lamellae. At higher CTAB concentrations, the increased content of polar phases may promote crystal perfection and lamellar rearrangement, contributing to the increase in *L*_c_ and *L* [46].

**Figure 6:**
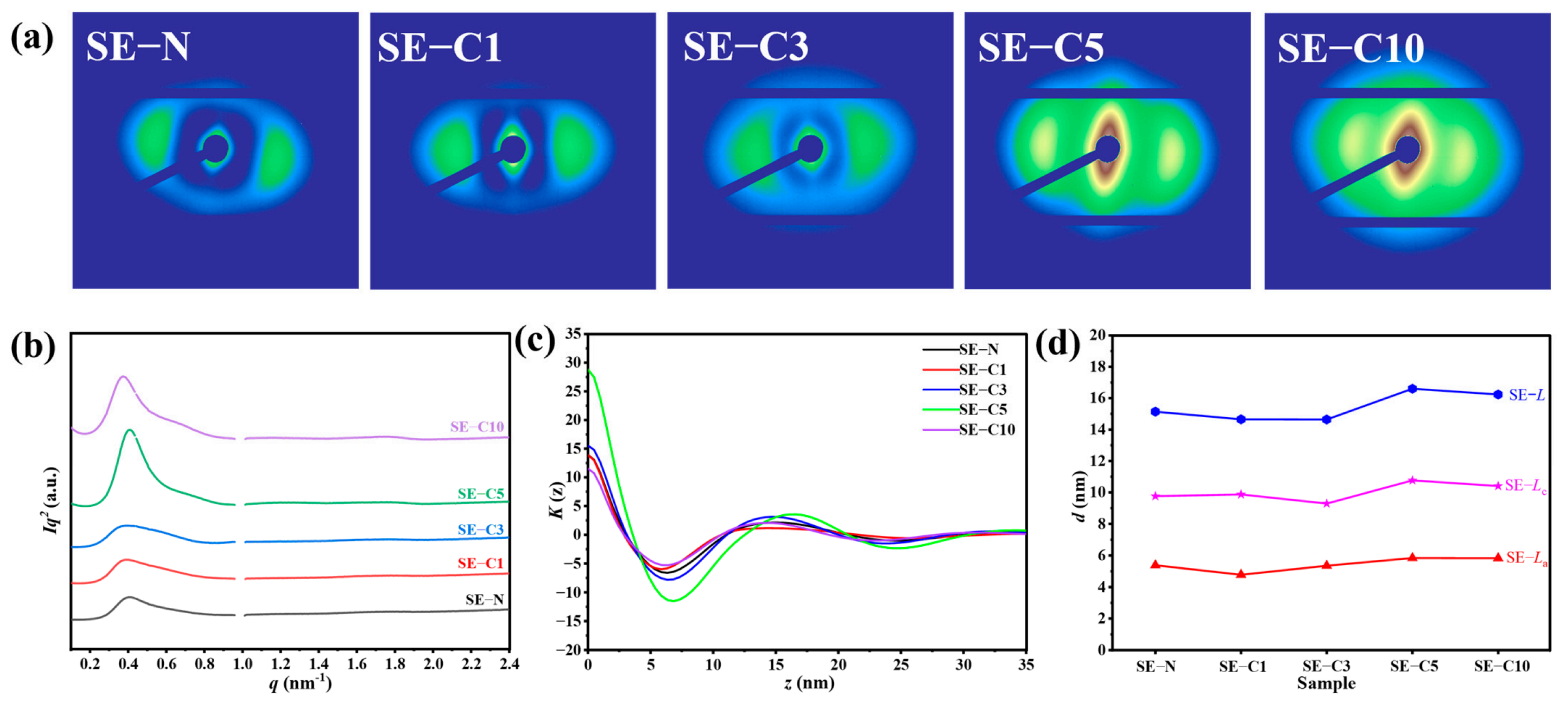
SAXS analysis of the solid-phase extruded PVDF/CTAB blends. (**a**) 2D-SAXS patterns of the solid-phase extruded samples with different CTAB contents. (**b**) Corresponding 1D-SAXS profiles. (**c**) One-dimensional electron density correlation functions of the extruded samples. (**d**) Comparison of the calculated crystalline lamellar thickness (*L*_c_), amorphous layer thickness (*L*_a_), and long period (*L*). Different colors represent different samples (SP-N, SP-C1, SP-C3, SP-C5, and SP-C10).

Compared with the die inlet samples, the SAXS patterns of the extruded samples exhibit a pronounced change. As shown in Figure 6a, after SPE, the 2D-SAXS patterns transition from nearly isotropic rings to a distinct spindle-shaped scattering concentrated along the equator, indicating preferential orientation of the lamellar normals perpendicular to the extrusion direction. For the neat extruded sample, weak anisotropic scattering is already discernible, indicating that the converging flow field initiates the deformation and rearrangement of the initially randomly distributed spherulites. Upon CTAB incorporation, the spindle-shaped scattering features become more pronounced, particularly in the extruded samples containing 5 and 10 wt% CTAB. This result suggests that strong ion–dipole interactions between PVDF and CTAB, together with the converging flow field, synergistically enhance lamellar orientation and ordering. Moreover, the pronounced meridional scattering streaks observed in SE-C5 suggests the possible formation of shish-kebab-like superstructures, reflecting a higher degree of molecular alignment and lamellar organization under the combined action of CTAB and the converging flow field.

The corresponding 1D-SAXS profiles are presented in Figure 6b. For the neat sample and samples containing less than 3 wt% CTAB, the scattering peak position remains nearly unchanged, indicating comparable lamellar long periods. Increasing CTAB content above 5 wt% causes the peak to shift toward lower *q* values with enhanced sharpness and intensity, suggesting improved nanoscale lamellar ordering induced by the synergistic effects of CTAB and the converging flow field. To further clarify the structural origin of this flow-induced increase in *L*, one-dimensional electron density correlation functions were calculated for the extruded samples, as shown in Figure 6c, and the corresponding quantitative results are summarized in Figure 6d. Since the variation in *L* is mainly governed by changes in lamellar thickness (*L*_c_), the increase in long period primarily originates from lamellar thickening. Notably, *L*_c_ remains nearly unchanged at low CTAB contents but increases significantly at 5 and 10 wt% CTAB, reaching a maximum for SE-C5. Compared with the die inlet samples, all extruded samples exhibit slightly increased *L*_c_ and *L* (Table S2), which can be attributed to flow-induced lamellar rearrangement during SPE and the associated limited lamellar thickening.

Taken together, appropriate CTAB incorporation strengthens ion–dipole interactions, which, combined with the converging flow field, promotes molecular orientation and lamellar reorganization. Among the samples investigated, the sample containing 5 wt% CTAB (SE-C5) exhibits the most favorable crystalline structural development.

**Figure 7:**
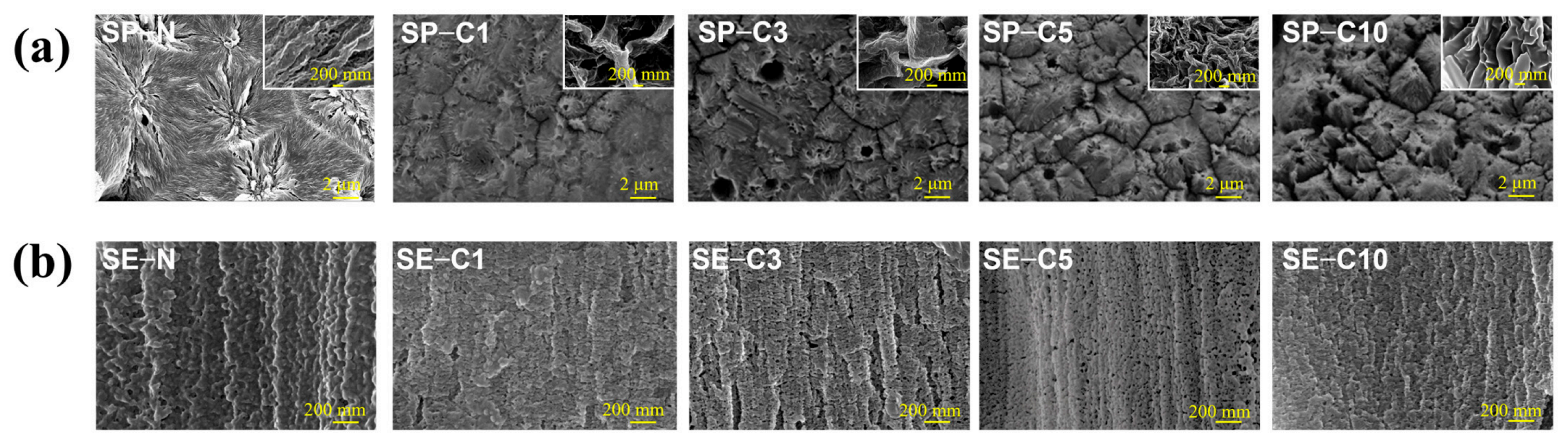
Surface SEM micrographs of chemically etched PVDF/CTAB blends from (**a**) the die inlet sample and (**b**) the extruded sample.

Although SAXS analysis reveals the evolution of lamellar orientation and periodicity, it cannot directly visualize the corresponding morphological changes in real space. Therefore, SEM was further employed to examine the surface morphology before and after SPE, providing direct evidence for the microstructural reconstruction. As illustrated in Figure 7a, the die inlet samples as a whole exhibit a randomly packed feature. In the neat die inlet PVDF, distinct radial spherulites are clearly discernible, indicating that without the die extrusion flow field, PVDF still predominantly crystallizes conventionally to form large-scale spherulitic morphologies. Upon the incorporation of CTAB, the strong ion–dipole interactions between PVDF and CTAB provide additional heterogeneous nucleation sites, resulting in refined crystalline domains and suppressed growth of large spherulitic structures. Specifically, the spherulitic boundaries in the die inlet samples with 1 and 3 wt% CTAB become blurred, accompanied by localized etching pits. Meanwhile, the die inlet samples with 5 and 10 wt% CTAB manifest rougher and more irregular blocky or granular morphologies. Nevertheless, all die inlet samples exhibit essentially isotropic spherulitic morphologies, indicating that CTAB-induced crystallization regulation alone cannot generate directional structural alignment, consistent with the SAXS observations.

Compared with their die inlet counterparts, the original spherulitic features after SPE become largely replaced by oriented lamellar bundles aligned along the extrusion direction, as illustrated in Figure 7b. In the neat extruded sample, the originally isotropic spherulite evolves into oriented lamellar stacks along the extrusion direction, resulting from the fragmentation of spherulites and subsequent lamellar rearrangement induced by the converging flow field. Upon the addition of CTAB, the oriented morphology is further intensified. The extruded samples with 3 and 5 wt% CTAB exhibit typical shish-kebab-like structures, characterized by continuous and directional lamellar arrangements along the extrusion direction. In particular, the 5 wt% CTAB sample displays a more uniform and ordered oriented morphology, indicating that an appropriate amount of CTAB synergistically cooperates with the converging flow to promote the formation of oriented lamellae [47]. In contrast, although the extruded sample with 10 wt% CTAB still maintains a discernible oriented morphology, its local structural uniformity decreases. This may be attributed to the excessively strong ion–dipole interactions, which restrict segmental mobility and hinder the cooperative response of molecular chains to the flow field, thereby limiting lamellar reorganization. This interpretation agrees well with the reduced flow-induced β phase increment and decreased crystalline lamellar thickness observed for SE-C10 compared with SE-C5.

### 3.4. Dielectric Properties of PVDF/CTAB Blends

**Figure 8:**
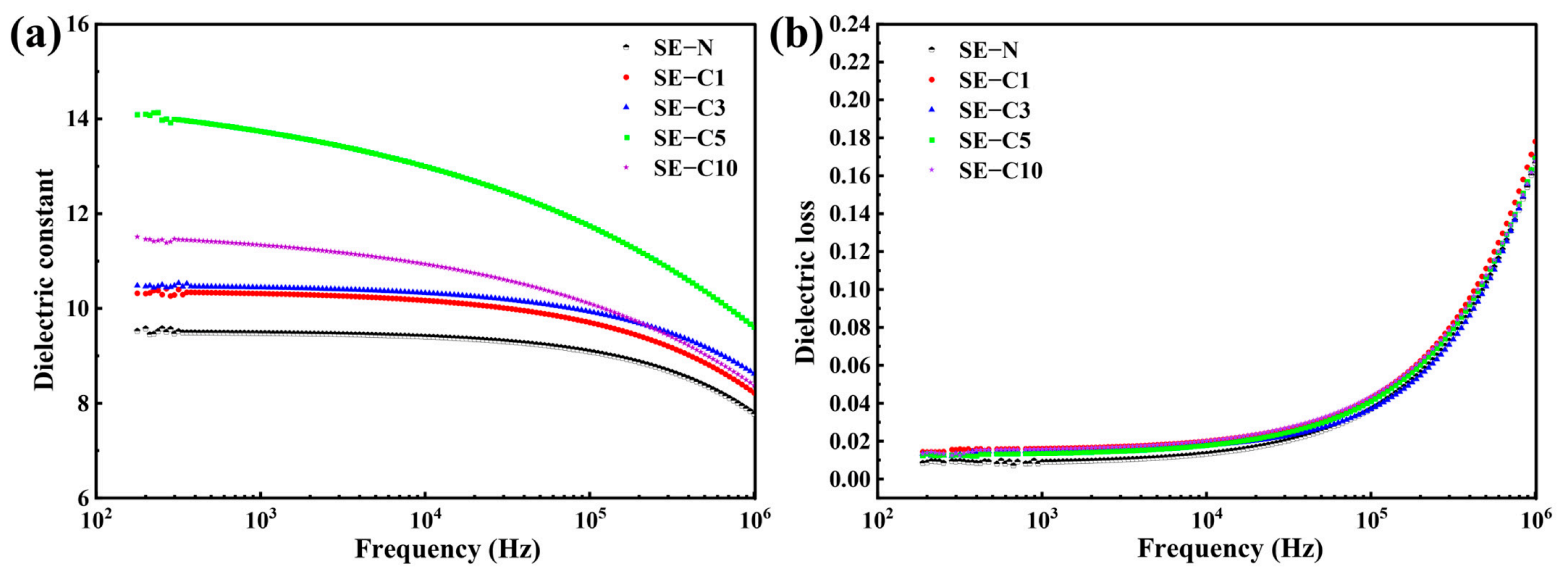
Frequency dependence of (**a**) dielectric constant and (**b**) dielectric loss for the solid-phase extruded (SE) PVDF/CTAB samples measured at room temperature.

To further understand the relationship between the hierarchical structure and dielectric performance of PVDF/CTAB blends, the frequency-dependent dielectric properties were measured at room temperature. As shown in Figure 8a, all samples exhibit a gradual decrease in dielectric constant with increasing frequency from 10^2^ to 10^6^ Hz. At low frequencies, the dielectric response mainly originates from dipolar and interfacial polarization, where polar groups and charge accumulation at phase interfaces can respond to the applied electric field [48]. With increasing frequency, dipolar polarization becomes progressively unable to follow the alternating electric field, resulting in a decrease in the dielectric constant.

With increasing CTAB content, the dielectric constant of the extruded samples increases progressively. Among all samples, SE-C5 exhibits the highest dielectric constant over the entire frequency range, indicating that the synergistic effects of moderate ion–dipole interactions and the converging flow field most effectively enhance the dielectric constant. This improvement can be attributed to the synergistic contribution of increased polar phases content and well-developed oriented lamellar structures. The higher β phase content increases the density of polar dipoles within the crystalline regions, while the enhanced lamellar ordering contributes to a more effective response. In contrast, although SE-C10 contains a higher CTAB content, its dielectric constant is lower than that of SE-C5, suggesting that excessive CTAB loading does not continuously improve dielectric performance. This behavior is consistent with the reduced molecular chain orientation and lamellar ordering observed in SE-C10, which weaken the overall polarization response [49]. To further evaluate the dielectric performance of SE-C5, its dielectric constant and dielectric loss at 1 kHz were compared with those of previously reported PVDF-based systems prepared using different processing strategies (Figure S4 and Table S3, Supplementary Materials). SE-C5 exhibits a dielectric constant within the upper range of the reported systems and the lowest dielectric loss among all listed PVDF-based systems.

As shown in Figure 8b, all samples exhibit an increasing dielectric loss with increasing frequency, particularly in the high-frequency region. The relatively low loss values indicate that CTAB incorporation enhances dielectric response without causing significant energy dissipation. Overall, the dielectric results demonstrate that dielectric performance depends not only on polar phase content but also on the optimization of the flow-induced hierarchical structure. The sample containing 5 wt% CTAB achieves the best balance between polar phase content and flow-induced lamellar reorganization.

### 3.5. Hierarchical Structural Evolution Mechanism of the PVDF/CTAB Blends During Solid-Phase Extrusion

**Figure 9:**
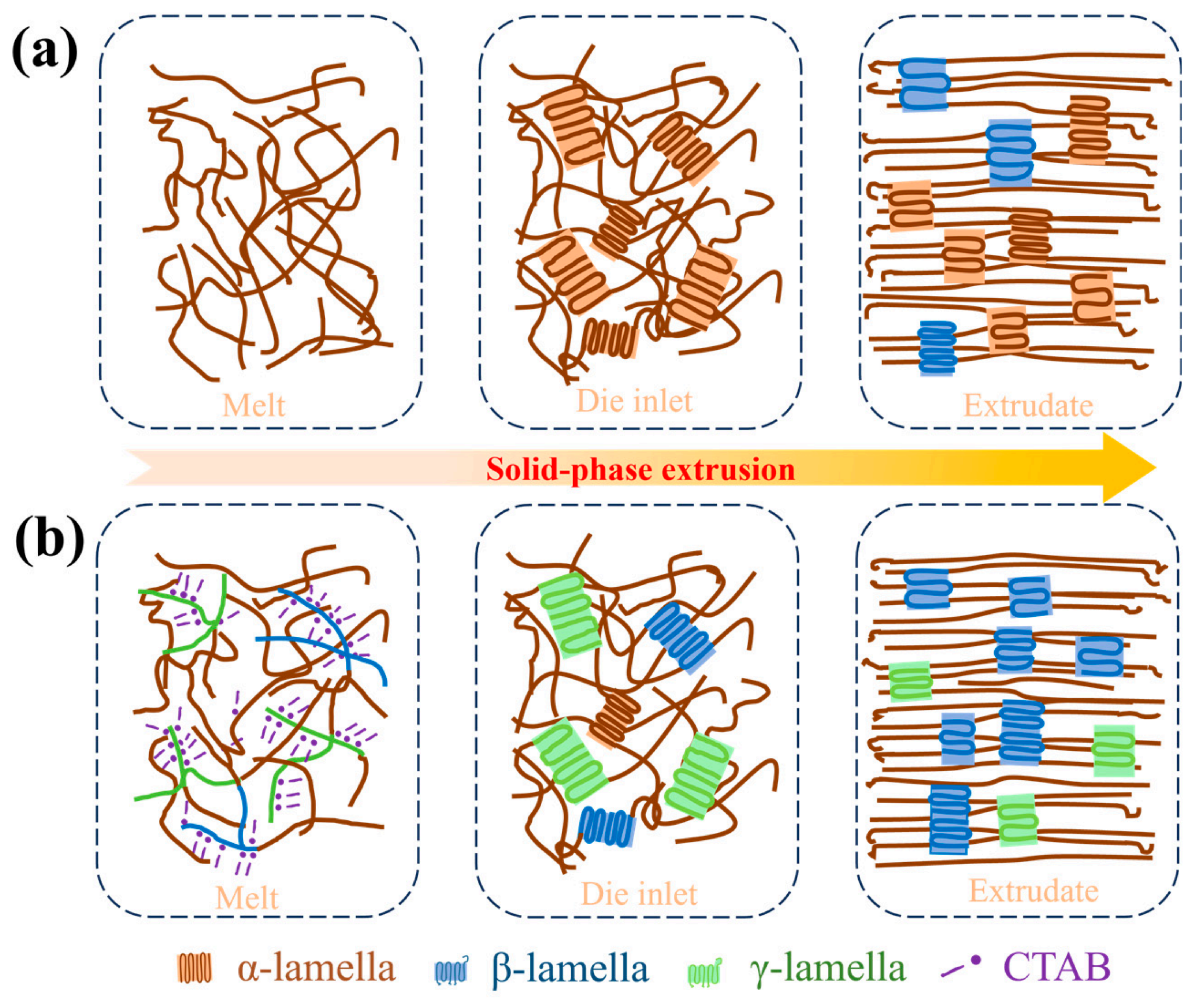
Schematic illustration of the crystalline phase evolution and oriented lamellar reorganization mechanism of (**a**) neat PVDF and (**b**) PVDF/CTAB blends during solid-phase extrusion.

Figure 9 summarizes the proposed structural evolution mechanism. CTAB regulates the initial crystalline phase structure through ion–dipole interactions, while the converging flow field subsequently drives molecular orientation and lamellar reorganization during SPE.

As shown in Figure 9a, in the molten state, PVDF chains are randomly distributed in a disordered random-coil state, and upon cooling, neat PVDF crystallizes predominantly into the nonpolar α phase. As the material enters the converging die during SPE, axial pressure applied by the plunger drives the solid billet through the progressively narrowing channel of the converging die. For neat PVDF, this combined flow field mainly induces spherulitic fragmentation, lamellar reorganization, and molecular alignment along the extrusion direction, leading to a progressively enhanced orientation while a substantial fraction of the original α phase is retained throughout the process, since the direct α-β phase transformation requires a considerably higher degree of chain extension than the γ-β pathway, which is not readily achieved under the flow field alone.

In contrast, as shown in Figure 9b, the incorporation of CTAB introduces strong ion–dipole interactions between the quaternary ammonium groups of CTAB and the C-F dipoles of PVDF chains, even in the molten state [50], which promotes locally ordered TTT sequences and favors the formation of polar phases. As a result, the die inlet PVDF/CTAB samples exhibit a coexistence of α, γ, and β phases, with the β phase fraction increasing progressively with CTAB content. Under the same converging flow field, however, the structural evolution of the PVDF/CTAB blends follows a markedly different pathway. The pre-existing γ phase generated by CTAB is considered to contribute to the subsequent development of β phases during extrusion. On one hand, the T_3_GT_3_G’ conformation of the γ phase represents a structurally closer precursor to the all-trans β configuration than the TGTG’ conformation of the α phase, corresponding to a lower energy barrier for the conformational change [51]. On the other hand, owing to its restricted chain mobility, the γ phase is more resistant to plastic flow than the α phase, allowing the converging flow field to transmit stress more effectively onto the crystalline skeleton rather than being dissipated through chain slippage, thereby further favoring the γ-β phase transformation [52]. As a result, the pre-existing γ phase generated by CTAB is transformed into the β phase through flow-induced conformational rearrangement, while the crystalline lamellae are simultaneously oriented along the extrusion direction, resulting in the highly oriented β phase, as shown in Figure 9b.

Overall, CTAB and extrusion affect different stages of PVDF structural evolution. CTAB promotes the formation of polar phases [53], particularly the γ phase, before extrusion, while the converging flow field further drives γ-β phase transformation and lamellar orientation during solid-phase processing [39].

## 4. Conclusions

This work systematically elucidates the evolution mechanism of PVDF crystalline structures regulated by the synergistic effects of CTAB-induced ion–dipole interactions and the converging flow field during solid-phase extrusion (SPE). The incorporation of CTAB alters the crystallization pathway of PVDF by suppressing the nonpolar α phase and promoting the formation of polar phases, with γ phase-enriched structures formed at low CTAB contents, which provide favorable precursors for the subsequent flow-induced γ-β phase transformation.

During SPE, the converging flow field further drives molecular chain stretching and orientation, promoting the γ-β phase transformation and inducing the evolution of crystalline morphology from randomly distributed spherulites into oriented lamellae along the extrusion direction. The synergistic action of CTAB and the converging flow field significantly enhances the β phase content and crystalline orientation of PVDF. Among the samples investigated, the sample containing 5 wt% CTAB exhibits the highest β phase content and the most favorable oriented crystalline structure. The optimized PVDF/CTAB blends exhibit enhanced dielectric performance, with an increased dielectric constant while maintaining low dielectric loss. These results demonstrate that the CTAB-assisted SPE strategy provides an effective route for constructing highly oriented β-PVDF films with improved dielectric properties.

## Figures and Tables

**Table 1:** Melting peak temperatures of the die inlet (SP) and solid-phase extruded (SE) PVDF/CTAB blends derived from DSC.

Sample	*T*_m1_ (°C)	*T*_m2_ (°C)
SP-N	170.7	— ^1^
SP-C1	170.1	175.2
SP-C3	167.8	174.4
SP-C5	166.9	176.2
SP-C10	163.2	174.6
SE-N	167.8	— ^1^
SE-C1	168.2	173.9
SE-C3	166.8	172.5
SE-C5	166.4	172.2
SE-C10	— ^1^	169.5

^1^ Only a single melting peak was observed for these samples.

## Data Availability

The original contributions presented in this study are included in the article. Further inquiries can be directed to the corresponding author.

## Supplementary Materials

The following supporting information can be downloaded at: https://www.mdpi.com/article/10.3390/polym18151901/s1, Figure S1: Cross-sectional schematic of the custom-designed solid–phase extrusion (SPE) apparatus; Figure S2:Relative crystalline phase fractions of the (a) die inlet and (b) extruded PVDF/CTAB samples as a function of CTAB content, quantified from FTIR spectra using Equations (2)–(5) in the main text; Figure S3: The calculated of *L*_c_, *L*_a_, and *L* according to the correlation function; Figure S4: Dielectric constant vs. loss (1 kHz) of SE-C5 and reported PVDF-based systems; Table S1: Lattice planes and corresponding *d*-spacings and peak positions for major crystalline phases of PVDF; Table S2: Flow-induced differences (Δ*L*, Δ*L*_c_, Δ*L*_a_ = SE − SP) for PVDF/CTAB blends at different CTAB contents; Table S3: Dielectric constant and loss (1 kHz) of SE-C5 compared with reported PVDF-based systems. References [37,39,54,55,56,57,58,59,60] are cited in the supplementary materials.

## Abbreviations

The following abbreviations are used in this manuscript:

PVDF	Poly(vinylidene fluoride)
CTAB	Cetyltrimethylammonium bromide
SPE	Solid-phase extrusion
2D-WAXD	Two-dimensional wide-angle X-ray diffraction
1D-WAXD	One-dimensional wide-angle X-ray diffraction
2D-SAXS	Two-dimensional small-angle X-ray scattering
1D-SAXS	One-dimensional small-angle X-ray scattering
ATR-FTIR	Attenuated total reflection Fourier-transform infrared spectroscopy
DSC	Differential scanning calorimetry
SEM	Scanning electron microscopy
