# Supplementary material for: Synergistic Flow Field and Ion–Dipole Interactions Enable γ-β Phase Transformation in Poly(vinylidene fluoride)
Source: Polymers (Basel). 2026 Aug 3;18(15):1901. doi: 10.3390/polym18151901 (PMC13468477; doi:10.3390/polym18151901)
Supplement: Supplementary file 1 [file polymers-18-01901-s001.zip › polymers-4436651-supplementary.pdf]

Supporting Information

Synergistic Flow Field and Ion-Dipole Interactions Enable γ-β Phase Transformation in Poly(vinylidene fluoride)

Qian Wang [1], Hong-Biao Yin [1], Hua-Jian Li [2], Xiang Bai [2], Fei Wang [1], Jianguo Liang [1], Guo-Zhen Ma [1], Jia-Yi Ren [1,*], Zhanchun Chen [1,*]

[1] *College of Mechanical Engineering, Taiyuan University of Technology, Taiyuan 030024, Shanxi, China*

[2] *Shanxi Energy Internet Research Institute, Taiyuan 030024, Shanxi, China*

* Correspondence:

renjiayi@tyut.edu.cn (J.-Y.R.)

chenzhanchun2003@163.com (Z.C.)

1.1 Solid Phase Extrusion Apparatus

The solid phase extrusion apparatus primarily consists of a hydraulic jack, a cylindrical barrel equipped with an external electrical heating jacket, a thermocouple for real-time temperature monitoring, a plunger for pressure application, and a replaceable converging die form (2 mm extruded thickness) mounted at the base of the barrel. As shown in Figure S1, the plunger applies a vertical pressure to the raw material loaded inside the barrel, while the heating jacket surrounding the barrel maintains the processing temperature, which is monitored in real time by a thermocouple.

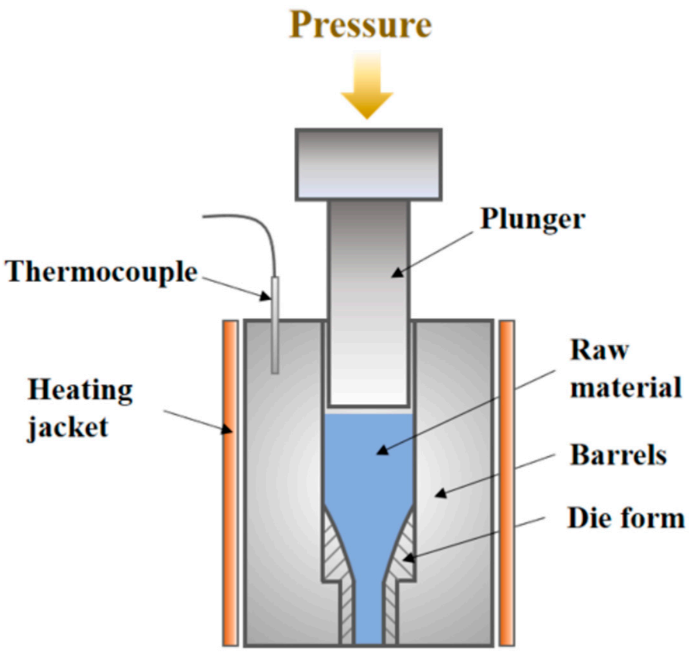

Figure S1. Cross-sectional schematic of the custom-designed solid phase extrusion (SPE) apparatus.

1.2 α, β, and γ Phase Contents Calculated from FTIR Spectra

Based on ATR-FTIR spectra, the relative contents of different crystalline phases were calculated. For the die inlet samples (Figure S2a), neat PVDF (SP-N) is dominated by the α phase, with only a minor β phase fraction and no detectable γ phase. Upon CTAB incorporation, the α phase fraction decreases markedly while the polar phases,

particularly the γ phase, increase substantially. The β phase content also gradually increases with increasing CTAB content, reaching 64.2% at 10 wt% CTAB (SP-C10). At CTAB loadings of 1-5 wt%, the γ phase remains the dominant polar phases, whereas the β phase becomes the predominant polar phases at 10 wt% CTAB, with a considerable amount of γ phase remaining, indicating the coexistence of β and γ phases.

For the extruded samples (Figure S2b), the converging flow field induces a pronounced evolution in the crystalline phase fractions. The α phase content decreases sharply across the series, while the β phase becomes the dominant crystalline component in all extruded samples, including the neat extruded sample (SE-N). The β phase content reaches a maximum of 80% in the sample extruded with 5 wt% CTAB, reflecting the optimal synergy between CTAB-induced polar phases and flow-induced chain stretching and orientation. Compared with the corresponding die inlet samples, the extruded samples exhibit a markedly lower γ phase fraction, indicating that the converging flow field promotes substantial γ-β phase transformation during extrusion. The slight decrease in β phase content to 69.8% at 10 wt% CTAB is consistent with the main text discussion, which may be associated with the excessively strong ion-dipole interactions at high CTAB loading that reduce the efficiency of cooperative stress transfer between chains and weaken flow-induced molecular chain orientation.

It should be noted that the crystalline phases fractions obtained from ATR-FTIR results are generally higher than those derived from WAXD results. This difference arises because ATR-FTIR predominantly probes the near-surface region, where the converging flow field induces the strongest chain orientation and β phase formation,

whereas WAXD reflects bulk-averaged information, consistent with the depth-dependent distribution of crystalline phases discussed in the main text (Section 3.1).

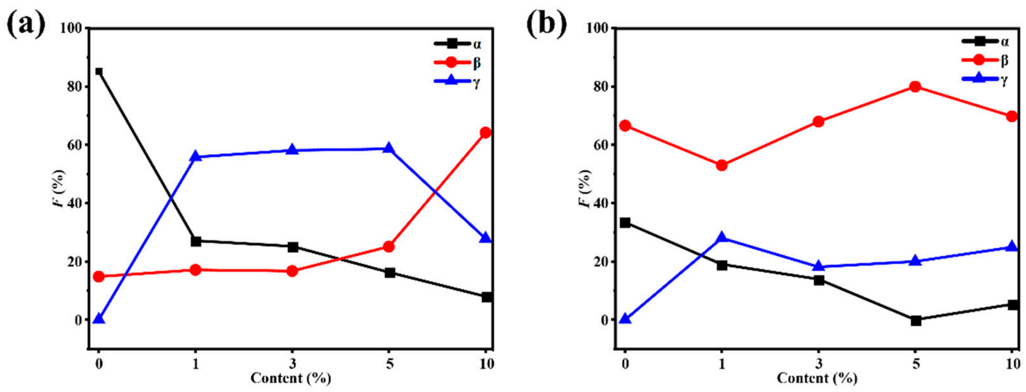

Figure S2. Relative crystalline phase fractions of the (a) die inlet and (b) extruded PVDF/CTAB samples as a function of CTAB content, quantified from FTIR spectra using equations (2)-(5) in the main text.

1.3 Assignment of the major reflection peaks for α, β, and γ phases

Table S1 summarizes the crystallographic parameters of the major crystalline phases of PVDF. The listed *d*-spacings and corresponding *q* values serve as references for the assignment of diffraction peaks discussed in the main text (Figures 2 and 3)[39].

Table S1. Lattice planes and corresponding *d*-spacings and peak positions for major crystalline phases of PVDF.

α phase			β phase			γ phase		
plane	*d* (nm)	*q* (nm^{-1})	plane	*d* (nm)	*q* (nm^{-1})	plane	*d* (nm)	*q* (nm^{-1})
100	0.496	12.67				100	0.495	12.68
020	0.482	13.04				020	0.479	13.12
110	0.441	14.25				110	0.440	14.28
			200	0.429	14.65			
			110	0.426	14.74	021	0.425	14.78
						111	0.390	16.10
021	0.334	18.83				022	0.332	18.92
111	0.319	19.69						
			001	0.256	24.54	131	0.256	24.54

1.4 Calculation of Lamellar Structural Parameters and Crystalline Orientation from SAXS/WAXD Analysis

The lamellar structural parameters were obtained from the one-dimensional electron density correlation function $K(z)$ derived from the SAXS profiles based on the two-phase model proposed by Strobl and Schneider[37]. The long period (L) was determined from the position of the first positive maximum of $K(z)$. Assuming a crystalline volume fraction higher than 50%, the amorphous layer thickness (L_a) was calculated as the intersection between the baseline and the extrapolated linear region of the self-correlation function. The crystalline lamellar thickness (L_c) was subsequently calculated using $L_c = L - L_a$. These parameters were used to analyze the effects of CTAB incorporation and solid phase extrusion on the lamellar structure evolution.

Figure S3 presents a representative correlation function with the characteristic structural parameters indicated. The calculated L_c, L_a and L values were used to evaluate the influence of CTAB incorporation and solid phase extrusion on the lamellar structural evolution, as discussed in the main text.

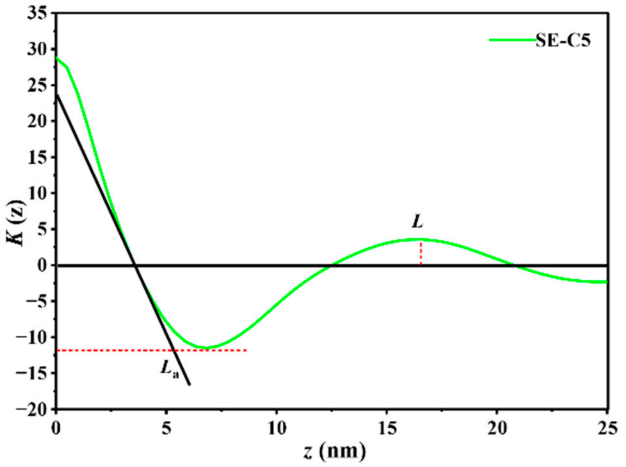

Figure S3. The calculated of L_c, L_a, and L according to the correlation function.

Table S2 decomposes the flow-induced change in the long period (ΔL) into its crystalline (ΔL_c) and amorphous (ΔL_a) contributions, allowing the relative roles of lamellar thickening and amorphous layer changes to be distinguished across different CTAB contents.

Table S2. Flow-induced differences (ΔL, ΔL_c, ΔL_a = SE - SP) for PVDF/CTAB blends at different CTAB contents.

CTAB content	ΔL = SE - SP (nm)	ΔL_c = SE - SP (nm)	ΔL_a = SE - SP (nm)
0 wt%	+0.50	+0.45	+0.05
1 wt%	+1.64	+1.74	-0.10
3 wt%	+1.44	+0.95	+0.49
5 wt%	+1.90	+1.41	+0.49
10 wt%	+0.73	+0.58	+0.15

1.5 Comparison of Dielectric Performance with Reported PVDF-Based Systems

To further evaluate the dielectric performance of the SE-C5 sample relative to previously reported PVDF-based systems, Figure S4 compares the dielectric constant and dielectric loss at 1 kHz of PVDF materials prepared using different processing strategies, while the corresponding data are summarized in Table S3. As shown in Figure S4, the SE-C5 sample exhibits a competitive dielectric constant together with the lowest dielectric loss among the reported systems, indicating a favorable balance between dielectric constant enhancement and loss suppression. The quantitative comparison presented in Table S3 further confirms this performance advantage. These results demonstrate that the synergistic effects of ion-dipole interactions and the converging flow field effectively optimize dielectric performance by enhancing polarization while suppressing dielectric loss.

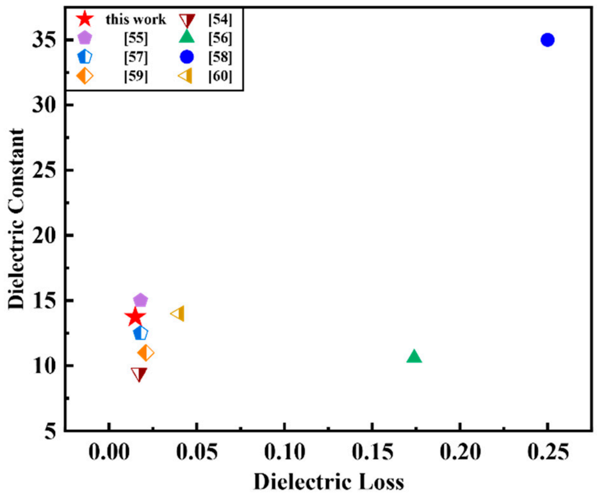

Figure S4. Dielectric constant vs. loss (1 kHz) of SE-C5 and reported PVDF-based systems.

Table S3. Dielectric constant and loss (1 kHz) of SE-C5 compared with reported PVDF-based systems.

Material System	Processing Method	Dielectric Constant (1kHz)	Dielectric Loss (1kHz)	Reference
PVDF/CTAB (5% CTAB)	Melt compounding followed by Solid Phase Extrusion (SPE)	13.74	0.015	this work
PVDF	Hot pressing under Cyclic Pulsating Pressure (CPP)	9.5	0.017	[54]
PVDF	Melt Extrusion	15	0.018	[55]
PVDF/IL (5 wt.% IL)	Fused Deposition Modeling (FDM 3D Printing)	10.6	0.174	[56]
PVDF	Double-step moulding (DS moulding)	12.5	0.018	[57]
PVDF/MMT/DES	Solution casting followed by isothermal crystallization	35	0.25	[58]
PVDF/TO@AO (1 wt%)	Solution casting followed by hot pressing	11	0.021	[59]
P(VDF-HFP)/ $FeCl_3{\cdot}6H_2O$ (4 wt%)	Solution casting	14	0.04	[60]

For references where numerical values at 1 kHz were not explicitly listed in the main text, data were extracted from the frequency-dependent dielectric spectra in the corresponding articles.

Supplementary References

39. Li, Y.; Tang, S.; Pan, M.-W.; Zhu, L.; Zhong, G.-J.; Li, Z.-M. Polymorphic Extended-Chain and Folded-Chain Crystals in Poly(Vinylidene Fluoride) Achieved by Combination of High Pressure and Ion–Dipole Interaction. *Macromolecules* **2015**, *48*, 8565–8573, doi:10.1021/acs.macromol.5b01895.

37. Strobl, G.R.; Schneider, M. Direct Evaluation of the Electron Density Correlation Function of Partially Crystalline Polymers. *J. Polym. Sci. Polym. Phys. Ed.* **1980**, *18*, 1343–1359, doi:10.1002/pol.1980.180180614.
54. Huang, Z.-X.; Wang, M.-M.; Feng, Y.-H.; Qu, J.-P. β-Phase Formation of Polyvinylidene Fluoride *via* Hot Pressing under Cyclic Pulsating Pressure. *Macromolecules* **2020**, *53*, 8494–8501, doi:10.1021/acs.macromol.0c01609.
55. Meng, N.; Ren, X.; Zhu, X.; Wu, J.; Yang, B.; Gao, F.; Zhang, H.; Liao, Y.; Bilotti, E.; Reece, M.J.; Yan, H. Multiscale Understanding of Electric Polarization in Poly(vinylidene fluoride)-Based Ferroelectric Polymers. *J. Mater. Chem. C* **2020**, *8*, 16436–16442, doi:10.1039/D0TC04310A.
56. Zhou, R.; Yang, H.; Zhao, L.; Wang, C.; Peng, C.; Wang, R.; Zhang, D.; Wang, X. Effects of Ionic Liquid Content on the Electrical Properties of PVDF Films by Fused Deposition Modeling. *Materials* **2024**, *17*, 9, doi:10.3390/ma17010009.
57. Brunengo, E.; Luciano, G.; Canu, G.; Canetti, M.; Conzatti, L.; Castellano, M.; Stagnaro, P. Double-Step Moulding: An Effective Method to Induce the Formation of β-Phase in PVDF. *Polymer* **2020**, *193*, 122345, doi:10.1016/j.polymer.2020.122345.
58. Yuan, M.; Wang, H.; Wang, T.; Yang, H.; Yuan, C. Synergistic Improvement in γ-Phase Content and Dielectric Properties of PVDF/Deep Eutectic Solvent/Montmorillonite Composite Films. *ACS Appl. Polym. Mater* **2023**, *5*, 2664–2673, https://doi.org/10.1021/acsapm.2c02241
59. Zhu, C.; Li, K.; Liu, X.; Li, Y.; Yin, J.; Hong, L.; Qin, Q. Enhanced Dielectric Performance in PVDF-Based Composites by Introducing a Transition Interface. *Polymers* **2025**, *17*, 137, doi:10.3390/polym17020137.
60. Yuennan, J.; Muensit, N.; Tohluebaji, N.; Chailad, W.; Yang, L.; Sukhawipat, N.; Ashraf, G.A.; Channuie, P. Tailoring Dielectric Properties and Crystallinity in Poly(Vinylidene Fluoride-Co-Hexafluoropropylene) Nanocomposites via Iron (III) Chloride Hexahydrate Incorporation. *Sci. Rep.* **2025**, *15*, 17810, doi:10.1038/s41598-025-02895-y.
